# Optimization of GOPS-Based Functionalization Process and Impact of Aptamer Grafting on the Si Nanonet FET Electrical Properties as First Steps towards Thrombin Electrical Detection

**DOI:** 10.3390/nano10091842

**Published:** 2020-09-15

**Authors:** Monica Vallejo-Perez, Céline Ternon, Nicolas Spinelli, Fanny Morisot, Christoforos Theodorou, Ganesh Jayakumar, Per-Erik Hellström, Mireille Mouis, Laetitia Rapenne, Xavier Mescot, Bassem Salem, Valérie Stambouli

**Affiliations:** 1 University Grenoble Alpes, CNRS, Grenoble INP, LMGP, F-38000 Grenoble, France; monica.vallejo-perez@grenoble-inp.fr (M.V.-P.); fanny.morisot@grenoble-inp.fr (F.M.); Laetitia.Rapenne@grenoble-inp.fr (L.R.); 2 University Grenoble Alpes, CNRS, DCM UMR 5250, F-38000 Grenoble, France; Nicolas.Spinelli@univ-grenoble-alpes.fr; 3 University Grenoble Alpes, Univ. Savoie Mont Blanc, CNRS, Grenoble INP, IMEP-LAHC, F-38000 Grenoble, France; christoforos.theodorou@grenoble-inp.fr (C.T.); mireille.mouis@grenoble-inp.fr (M.M.); xavier.mescot@grenoble-inp.fr (X.M.); 4 KTH Royal Institute of Technology, Department of Electronics, School of Electrical Engineering and Computer Science, Electrum 229, SE-164 40 Kista, Sweden; Ganesh.Jayakumar@imec.be (G.J.); pereh@kth.se (P.-E.H.); 5 University Grenoble Alpes, CNRS, CEA/LETI Minatec, Grenoble INP, LTM, F-38054 Grenoble, France; bassem.salem@cea.fr

**Keywords:** silicon nanowires, nanonet, field effect transistors, GOPS-based functionalization, UV-assisted functionalization, aptamer grafting

## Abstract

Field effect transistors (FETs) based on networks of randomly oriented Si nanowires (Si nanonets or Si NNs) were biomodified using Thrombin Binding Aptamer (TBA–15) probe with the final objective to sense thrombin by electrical detection. In this work, the impact of the biomodification on the electrical properties of the Si NN–FETs was studied. First, the results that were obtained for the optimization of the (3-Glycidyloxypropyl)trimethoxysilane (GOPS)-based biofunctionalization process by using UV radiation are reported. The biofunctionalized devices were analyzed by atomic force microscopy (AFM) and scanning transmission electron microscopy (STEM), proving that TBA–15 probes were properly grafted on the surface of the devices, and by means of epifluorescence microscopy it was possible to demonstrate that the UV-assisted GOPS-based functionalization notably improves the homogeneity of the surface DNA distribution. Later, the electrical characteristics of 80 devices were analyzed before and after the biofunctionalization process, indicating that the results are highly dependent on the experimental protocol. We found that the TBA–15 hybridization capacity with its complementary strand is time dependent and that the transfer characteristics of the Si NN–FETs obtained after the TBA–15 probe grafting are also time dependent. These results help to elucidate and define the experimental precautions that must be taken into account to fabricate reproducible devices.

## 1. Introduction

Thanks to the combination of unique tunable electrical properties with a high surface to volume ratio, Si nanowires (Si NWs) show an extreme sensitivity of their charge carriers to any modification of potential at their surface. They are thus promising building blocks for field effect transistor (FET)-based sensors for gas and biomolecule electrical detection. For these reasons, extensive research work has been performed on Si NW–FETs for electrical detection of various charged biomolecules such as DNA. This is attested by reviews [1,2,3], summarizing advances on Si NW–FETs made of parallel Si NW arrays with NWs patterned by top-down techniques such as conventional photolithography, electron beam lithography [4], sidewall transfer lithography [5] and nano-imprinting approaches. In all these works, the resulting DNA detection limit varies between 0.1 fM [6] to 1 nM [7], depending on numerous parameters (Si NW size, NW doping, functionalization method, distance between DNA and NW surface, ionic medium, etc.). However, obtaining NWs from top-down fabrication techniques remains complex, time consuming and costly.

To overcome these drawbacks, an alternative approach consists of transferring bottom-up grown single Si NWs on a substrate and integrating them in a FET structure. Few results are published on DNA electrical detection with such approach. Examples can be found in [8] and references herein for single NW devices. In these cases, one advantage is the possibility to benefit from Si NWs with small diameters (less than 10 nm). However, the device fabrication requires a precise alignment and positioning of the Si NWs leading to integration issues. More results can be found with different nanomaterials such as graphene nanonets (GNN) [9] or carbon nanotube (CNT) networks [10]. Nevertheless, silicon is one of the most widely used materials, and its success is due to the fact that it is low cost and highly compatible with the Si-based CMOS technology [11].

Another FET-type integration solution is the Si nanonets (Si NN), which are 2D networks of randomly oriented Si NWs, which can be transferred on a wide range of substrates and notably CMOS readout circuits. To our knowledge, apart from the results presented by our group about Si NN in view of DNA detection, ranging from Si NN fabrication [12], Si NN integration in FET devices to provide Si NN–FETs [13] and Si NN–FETs functionalization for DNA sensing [14], there have not been any other related works published yet.

With Si NN–FETs, sensing performances for reliable and reproducible electrical detection of biomolecules, such as DNA sequences, strongly depend on many physical factors located at different levels. First, at the level of the Si NWs, their intrinsic characteristics are important: semiconductor properties (doping, mobility, etc.) and morphology (length, diameter, etc.). Moreover, the electrical and chemical stability of the Si NWs are crucial, and passivation by an oxide layer more stable than native SiO_2_ is required. Notably, we have shown that Si NN–FET devices passivated with an 8 nm thick Al_2_O_3_ layer exhibited a high electrical stability throughout a study conducted over several months [13].

Second, at the Si NN level, the electrical properties are governed by the percolation theory, according to which the current flows via conducting paths involving Si NWs and NW/NW junctions. This was successfully performed thanks to an optimized sintering process [15]. Obviously, the NN density and the number of NW/NW junctions are also important factors.

Third, at the NN-FET level, an important factor is the geometry of the NN channel: length and width. All these factors have been optimized to provide reproducible devices presenting transfer characteristic curves with high I_on_/I_off_ ratio. Consequently, by controlling all steps of Si NN–FET fabrication for optimized electrical characteristics, we can establish Si NN–FETs as promising devices for electrical biosensing.

In view of this application field, this study is part of a wider project whose objective is to elaborate a biosensor to electrically detect the thrombin, a protein involved in the physiology of blood clots. This protein has been chosen as a model protein as the thrombin binding aptamer (TBA–15) is well known. Aptamers are short RNA, single-stranded DNA, or synthetic XNA molecules that can fold into stable and unique three-dimensional structures through intermolecular interactions such as electrostatic and van der Waals interactions, stacking of aromatic rings or hydrogen bonds. Aptamers can interact with their targets with high specificity and affinity [16,17]. TBA–15 is a specific G-quadruplex oligonucleotide probe for detection of thrombin (K_d_ ~35–100 nM [18]), which is a protein that plays a physiological role in regulating hemostasis and maintaining blood coagulation cascade [19]. This work focuses on the immobilization of thrombin binding aptamer (TBA–15) and its impact on electrical properties of Si NN–FETs as a first step towards the thrombin biosensor. In particular, special attention was paid to the impact of the selected materials, the grafted DNA sequences and the solutions used on the electric properties of the Si NN–FETs, in order to identify the experimental conditions necessary for a good reproducibility of the devices and experiments. For this purpose, a suited chemical functionalization process is required to obtain a homogenous distribution of strongly grafted TBA–15 probes on the Si NN surface, which is crucial to facilitate the efficient recognition with the target molecules to be detected. Then, the impact of TBA–15 grafting on Si NN–FET electrical properties was studied. To reach this objective and ensure the proper conduct of experiments, part of the experimental work includes the hybridization of TBA–15 with its complementary DNA sequence. With the aim of controlling the behavior and reproducibility of the biosensor as well as possible, it is important to control the biomodification process and to carefully characterize the NN–FETs after the biomodification process.

Common methods for surface biomodification of Si NWs use organosilanes to introduce various functional groups on the Si NW surface. One of the frequently implemented organosilanes for immobilization of amino-terminated DNA probes is an aminosilane: (3-Aminopropyl)triethoxysilane (APTES). It allows for the obtainment of an amino-functionalized Si NW surface. Another interesting option is an epoxy silane: (3-Glycidyloxypropyl)trimethoxysilane (GOPS), which allows the formation of an epoxy ended surface. By contrast with the amine-terminated surface, which requires the use of a cross-linker, such as a dialdehyde, to make the covalent binding of NH_2_-terminated DNA probes on the surface, the epoxy-terminated surface allows a direct covalent attachment of NH_2_-terminated DNA probes on the surface via ring-opening reaction on the epoxy group, thus avoiding the cross-linker step. We have previously implemented a new functionalization procedure using GOPS deposited in vapor phase, and reported the results first on Si NNs [20] and second on Si NNs, after their integration in FETs for electrical DNA hybridization detection [14]. Both of these silanization processes have pros and cons. Here, our aim is to determine which is the best process for TBA–15 grafting on Si NN–FETs in view of electrical biosensing and how to optimize it.

For the work presented in this paper, first, we have studied the impact of both APTES and GOPS silanization processes on the finalized Si NN–FET devices. This study has led us to select the GOPS based process. Second, in order to optimize TBA–15 probe grafting on the epoxy-terminated Al_2_O_3_ passivated Si NNs, we have preliminarily investigated the influence of UV irradiation on the TBA–15 grafting based on some previous studies, which showed the potential of UV cross-linking to enhance the grafting of oligonucleotides [21,22,23]. However, in such UV-assisted biofunctionalization processes, the DNA is directly exposed to radiation during the grafting step. This exposition can cause mutations that are directly related to DNA damage when UV-C light (UVC, λ = 254 nm) is used [24]. For this reason, we decided to work with UV-A light (UVA, λ = 365 nm) that carry less energy than UVC, by analyzing the influence of the use of UV light on GOPS silanized surfaces, just before and just after TBA–15 drop casting for different exposure times. Based on standard (contact angle, fluorescence) and advanced (atomic force microscopy (AFM) and energy dispersive X-ray spectroscopy in scanning transmission electron microscopy (STEM EDS)) characterization techniques, we demonstrate the successful attachment of TBA–15 probe and the better homogeneity thanks to UV-assisted GOPS based functionalization on Si NN–FETs. Third, after TBA–15 grafting optimization, we have studied the impact of the aptamer grafting on the electrical characteristics of the Si NN–FETs with different geometries. We demonstrate that the results are highly dependent on the experimental protocol, and thus provide an outline for their proper definition. We also found that the transfer characteristics obtained after probe grafting and the hybridization capacity are time dependent.

## 2. Materials and Methods

### 2.1. Fabrication of the Aptamer-Based Si NN–FETs

#### 2.1.1. Elaboration of Al_2_O_3_ Passivated Si NN–FETs

**Figure 1:**
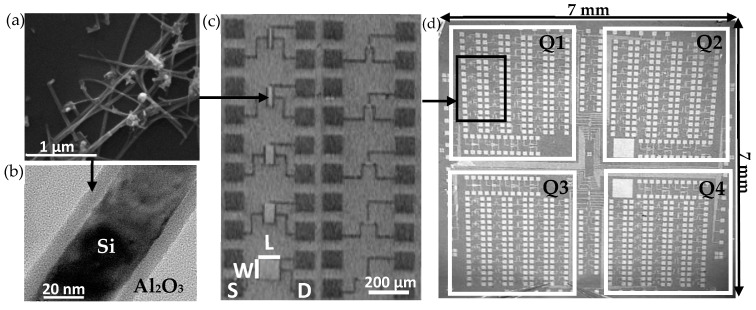
(**a**) SEM image of the Si nanonet (NN) after transfer on heavily doped Si substrate covered with a 200 nm thick Si_3_N_4_. (**b**) High-resolution TEM (HRTEM) image displaying an Al_2_O_3_ passivated Si nanowire. (**c**) Optical image of Al_2_O_3_ passivated Si NN field effect transistors (FETs) presenting different channel geometries. On the image, W, L, S and D refer to channel width, channel length, source and drain, respectively. (**d**) Optical image of the resulting chip. Q1, Q2, Q3 and Q4 denote the different quarters of the device.

The fabrication of Si NN–FETs has already been reported by our group [13,14,25,26]. The process begins with the formation of a randomly oriented network from a suspension of Si nanowires elaborated by chemical vapor deposition (CVD) using a vapor–liquid–solid (VLS) mechanism [12]. The nanowires have an average diameter of 39 nm ± 7 nm, an average length of 7 µm ± 3 µm and present a p-type behavior with an estimated doping level of about 10^16^ at·cm^−3^. The density of the Si nanowires in the suspension is controlled by absorption spectroscopy at 400 nm. Subsequently a vacuum filtration of the suspension is carried out on a nitrocellulose membrane, which is transferred to a 1 cm × 1 cm heavily doped Si substrate covered with a 200 nm thick Si_3_N_4_ layer, which acts as the oxide gate of the Si NN–FET. The nitrocellulose membrane is then dissolved by an acetone bath, giving rise to a network of randomly oriented Si nanowires on the substrate (Figure 1a).

The nanonet density ($D_{NN}$) (NWs·cm^−2^) is directly related to the filtered volume ($V_{filtered}$) and can be calculated by Equation (1), when absorbance at 400 nm is equal to 0.06 [14]:

(1) $$D_{NN}=2.34\times V_{filtered}\times 10^{6}\;$$

In this work, a volume of 18 mL was filtered, resulting in a density of 42 × 10^6^ NWs·cm^−2^.

The native oxide covering the NN is removed by using a HF/NH_4_F (6:1) solution (buffer oxide etching, BOE (Chimie Tech Services, France)), and immediately the whole is subjected to a sintering at 400 °C under nitrogen, with the aim of creating conductive paths between the nanowires. This process improves the electrical properties of the Si NN FETs by two orders of magnitude and makes them insensitive to oxidation in the air [15]. The NN is then passivated with an 8 nm thick alumina layer deposited by atomic layer deposition (ALD, Fiji 200 from Ultratech) (Figure 1b).

Patterning of Al_2_O_3_ passivated Si NN–FETs is carried out by the combination of wet (BOE) and dry (SF_6_) etching in order to create different Si NN channel geometries varying from 5 µm to 200 µm for the length (L), and from 10 µm to 100 µm for the width (W). Subsequently, by combining photolithography and lift off, Ni/Au source (S) and drain (D) metal contacts are deposited by Electron Beam Evaporation (MEB550 from Plassys). The final step consists of an annealing at 400 °C under N_2_, called silicidation, which aims to create a low resistive phase (NiSi) between the Si from the NN and the Ni from the metal contacts. This step significantly improves the electrical properties of the Al_2_O_3_-passivated Si NN–FETs [25]. Figure 1c shows some of the device geometries obtained. The resulting 7 × 7 mm^2^ chips contain about 180 devices (Figure 1d) which are ready for the biofunctionalization steps. Each chip is composed of 4 similar quarters, each containing 45 devices with different geometries (W–L combinations).

#### 2.1.2. Oligonucleotides

Several oligonucleotide sequences, described in Table 1, were used. Except TBA–15 which was synthesized at DCM on a DNA synthesizer (ABI 3400, Foster city, CA, USA) using phosphoramidite protocol, the other sequences were purchased from Biomers (Ulm, Germany). The probes were grafted on the chip thanks to their NH_2_ termination. As our aim is to design a thrombin biosensor, the thrombin binding aptamer (TBA–15), with a six thymines spacer at 3’ end, was the principal probe. In addition, a second probe (Sequence 2) was also grafted on the chip to study the impact of the DNA sequence on the SiNN–FET properties. Lastly, the complementary DNA sequences to the TBA–15 (cTBA) and Sequence 2 (c-Sequence 2) probes were used as the target molecule for control purposes using epifluorescence microscopy. To this end, the targets were labeled with a cyanine fluorophore (Cy3). According to the probe used, each chip was divided into four quarters (Figure 1d). Two quarters (top-right, Q2 and bottom-right, Q4) were functionalized with TBA–15 probe, whereas one quarter (bottom-left, Q3) was functionalized with Sequence 2 probe. The last quarter (top-left, Q1) was not functionalized with DNA probe, but was submitted to every step of the functionalization process, except that no DNA probe was added in the grafting solution.

#### 2.1.3. DNA Probe Grafting and Hybridization

The grafting process of the DNA probes, TBA–15 and Sequence 2 (see Table 1), was carried out using two different organosilane deposition-based protocols previously reported: an amino-silane (APTES) protocol [27,28] and an epoxy-silane (GOPS) protocol [20]. The functionalization process consists of three main stages (detailed protocols are provided in Appendix A and Appendix B). Initially, an activation of the surface was carried out by the creation of pending hydroxyl groups thanks to an exposure to an O_2_/air plasma. Silanization was then performed by depositing either APTES or GOPS in vapor phase on the substrate. Finally, covalent linking of DNA probes was achieved with the help of a cross-linker (glutaradehyde) in case of APTES, or directly in case of GOPS, for which the epoxy group reacts with the amine-modified DNA dissolved in phosphate buffer (sodium phosphate dibasic). A droplet of 7 µL of the grafting solution was deposited on each quarter of the chip (Figure 1d). For each sequence, the concentration of DNA probes in the droplet was 10 µM, except for the Q1 quarter, where no DNA probe was added in the grafting solution.

After the probe grafting step, the biosensor is ready for hybridization. As a consequence, the chip was then exposed to DNA targets: c-TBA or c-sequence 2 (see Table 1). The concentration of the DNA target was 2 µM in PBS (sodium phosphate dibasic, sodium phosphate monobasic, KCl, NaCl), and the volume used was 28 µL. After the reaction time at 42 °C, the samples were rinsed and dried. The results were subsequently analyzed by fluorescence microscopy, image processing and by electrical measurements, as explained below.

As part of this work, the GOPS-based grafting process was optimized using UV radiation. This was preliminarily implemented on 1 cm × 1 cm Si wafers covered with a 100 nm thick SiO_2_ layer. Si/SiO_2_ GOPS silanized surfaces were exposed to 365 nm UV-A light (39 mW/cm^2^) using a UV–LED masking system (UV–KUB 2 by Kloé, France). For this optimization study, various experimental conditions were chosen: three increasing exposure times (10, 20 and 40 min) in the case of UV exposure just before TBA–15 probe drop casting, and in the case of UV exposure just after TBA–15 probe drop casting. In order to analyze the effect of UV exposure on DNA grafting, the hybridization with the DNA target (c-TBA strand) was carried out and the results were evaluated by epifluorescence microscopy, in order to determine the parameters that allow an optimal grafting of the TBA–15 probe on the surface. Once the functionalization process was optimized on Si/SiO_2_ substrates, the protocol was evaluated on: (i) Si (100) wafers covered with a 100 nm thick SiO_2_ layer and 8 nm thick Al_2_O_3_—the latter is the same layer used to passivate the Si NN in our devices, and (ii) Al_2_O_3_ passivated Si NN–FETs.

### 2.2. Characterization Techniques

#### 2.2.1. Fluorescence

Thanks to the Cy3 fluorophore, the grafting process was verified by DNA hybridization using an Olympus BX41M fluorescence microscope (Olympus, Rungis, France) coupled with a 100W mercury lamp. The fluorescence intensity of the eight-bit jpg images was analyzed by the analysis software ImageJ (1.44p, Wayne Rasband, National Institutes of Health, USA), which allows quantifying the intensity through a gray scale for each image, having previously subtracted the background signal for each fluorescent spot.

#### 2.2.2. Wettability

Water contact angle measurements on Si/SiO_2_ silanized surfaces were performed on sessile droplets using a drop shape analysis system DSA10 Mk2 (Kruss GmgH, Hamburg, Germany). Three measurements were made for each sample, and the result reported corresponds to the average value.

#### 2.2.3. Atomic Force Microscopy (AFM)

AFM analyses were carried out on Al_2_O_3_ passivated Si NN FETs before and after the main stages of the biofunctionalization process by means of a Dimension Icon AFM purchased from BRUKER (Palaiseau, France). Images were acquired in air using non-contact/tapping mode, with a BRUKER AFM probe, model RTESP-300. All AFM experiments were performed with a scan size of 820 nm, a scan rate of 0.543 Hz and were taken with 256 samples/line × 256 lines. The surface topography, roughness and size of the observed structures on various locations of the Al_2_O_3_ passivated Si NN–FETs were explored by image processing using the software Gwyddion (2.56, Department of Nanometrology, Czech Metrology Institute, Brno, Czech). All data were extracted from the height AFM images, and, when necessary, images were processed by flattening. For root mean square (RMS) roughness, three different zones on the Al_2_O_3_-covered substrate with the same area (100 nm × 100 nm) were analyzed, and the result presented corresponds to the average value for each sample. The dimension of biomolecules (height) was extracted by line profiles of the molecules, and the value presented reflects the average of 10 measurements.

#### 2.2.4. Transmission Electron Microscopy (TEM)

TEM analysis was performed on biofunctionalized Al_2_O_3-_passivated Si NN FETs by scratching the surface of the chip using a diamond tip and spreading the obtained material on a holey carbon-coated TEM support cupper grid. High-resolution TEM (HRTEM) was performed on a JEOL 2010 LaB6 200KeV (JEOL, Japan) TEM. STEM–EDS spectra and maps of Si NNs were collected with a JEOL SDD Centurio detector that had a large solid angle of up to 0.98 steradians; incorporated in the JEOL 2100F field-emission-gun scanning electron microscope operating at 200 kV and having a 0.2 nm resolution in the scanning mode.

#### 2.2.5. Electrical Characterization

The current–voltage electrical characterization of the devices was acquired through a three-probe Karl Süss station (Dresden, Germany) controlled by a HP4155A (Yokogawa-Hewlett-Packard, Ltd., Tokyo, Japan) parameter analyzer before and after the main steps of the biofunctionalization process. All measurements were carried out in a dry and dark environment, at room temperature and ambient conditions, using the same integration time (medium).

## 3. Results and Discussion

### 3.1. APTES Based Functionalization Related Issues

**Figure 2:**
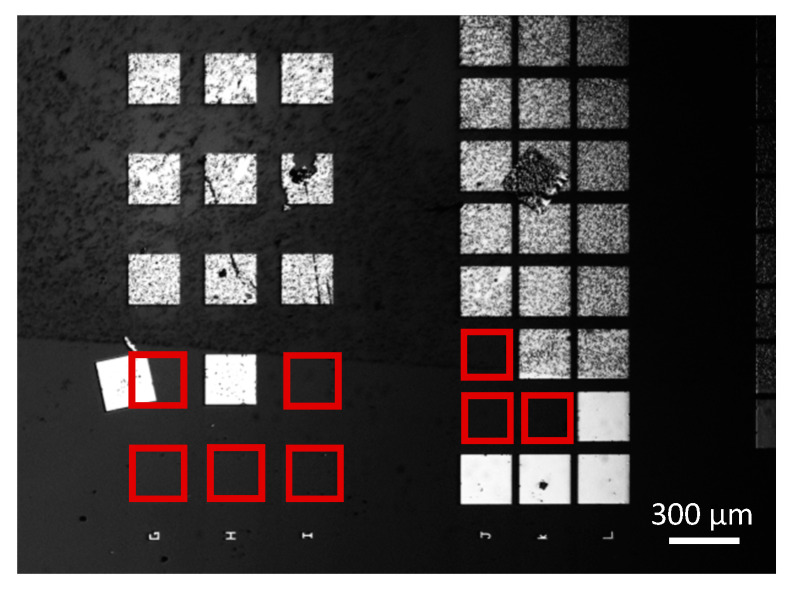
SEM image of functional chips consisting of Si NN–FETs evidencing metal contact delamination due to the reduction reaction step related to the APTES-based functionalization protocol.

The aminosilane-based functionalization protocol has been widely studied in the literature, and we recently optimized it for the vapor phase deposition of APTES [27,28]. When carried out on full wafer—with or without Si nanonets—this functionalization process works properly with intense and highly homogeneous fluorescence [27]. However, when the process is performed on the functional chips consisting of NN-FETs, various issues occur in the very last step of the functionalization. During the reduction of imine bonds into amine ones by NaBH_4_ (see Appendix A), metallic contact delamination was observed (see Figure 2) due to H_2_ bubbles generated as a by-product of imine reduction reaction. Several tracks have been followed to solve this issue but without success. First, we changed the position of the chip during the reduction step in order to analyze the effect of the type of exerted force (normal or tangential) by the bubbles on the metal contacts. Second, we worked on the optimization of the NaBH_4_ solution concentration and duration step—with the important result that reducing the concentration of NaBH_4_ from 0.09 M to 0.01 M and reaction time from 1 h to 15 min during the imine reduction step maintains a very efficient DNA grafting protocol. As a consequence, the APTES-based protocol should be used only with metal contact encapsulated devices. These technological developments are currently under investigation. For the present study, taking into account that metal contacts are not encapsulated and with the aim to develop a shorter protocol safe for the metallic pads, we focused on the optimization of the DNA probe grafting thanks to GOPS silanization.

### 3.2. Optimization of DNA Grafting by UV-Assisted GOPS-Based Functionalization

**Figure 3:**
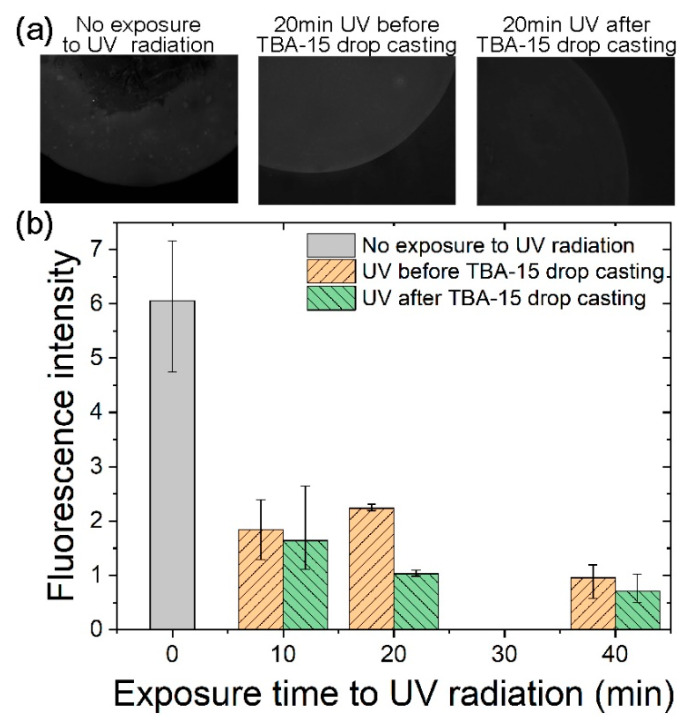
Analysis of fluorescence intensity for TBA–15 grafting and hybridization by means of GOPS-based functionalization with and without exposure to UV light. (**a**) Typical epifluorescence microscopy images of GOPS silanized Si/SiO_2_ (100 nm) substrates in different cases regarding UV treatment (no UV exposure, 20 min UV exposure before and after TBA-15 drop casting) and after DNA grafting and hybridization. (**b**) Fluorescence intensity, as measured on fluorescence micrographs, as a function of exposure time to UV radiation. For all conditions, three fluorescent spots on the substrate were analyzed giving an average fluorescence intensity value and error bars corresponding to the minimum and maximum fluorescence intensity found for each sample.

GOPS silanized Si/SiO_2_ (100 nm) substrates were exposed to 365 nm UV light (39 mW/cm^2^) for three increasing exposure durations (10, 20 and 40 min), before or after TBA–15 probe drop casting. Subsequently, the hybridization with complementary fluorescent target was carried out and analyzed using fluorescence microscopy. For all conditions, three fluorescent spots on the substrate were analyzed giving an average fluorescence intensity value and error bars corresponding to the minimum and maximum fluorescence intensity found for each sample (Figure 3).

On the corresponding histogram (Figure 3), it is observed that for each duration, the intensity of fluorescence is higher when UV exposure is carried out before TBA–15 probe drop casting. When the UV exposure is applied after the TBA–15 drop casting, the fluorescence intensity decreases as the exposure time increases. Both observations suggest that the effect of UVA on DNA probes is not negligible and that the only way to ensure the integrity of the DNA strands is to place the UV exposure before the DNA probe drop casting.

It is noted that the highest fluorescence intensity is obtained without UV exposure. However, the fluorescence is not homogeneously distributed over the spot as shown on the corresponding image and confirmed by the important error bar (Figure 3). Thus, the use of UV irradiation reduces the mean fluorescence intensity but increases drastically the homogeneity particularly for the 20 min exposure. To conclude, the best result—high fluorescence intensity and good homogeneity—is obtained for a UV exposure time of 20 min before TBA–15 probe drop casting.

To enlighten the improvement in the homogeneity, contact angle measurements were carried out on GOPS-silanized surfaces, which were exposed for 20 min or not to UV radiation. The results gave a value of 78.4° (standard deviation σ = 3.0°) for the surface that was not exposed to UV, and 68.8° (σ = 1.4°) for the irradiated surface, meaning that UV radiation makes the surface more hydrophilic. Such an increase in hydrophilicity is probably responsible for the observed improvement, as TBA–15 probe dissolved in a saline solution at the moment of the grafting can reach more easily the surface when the latter is more hydrophilic.

This UV-optimized GOPS-based functionalization protocol was subsequently evaluated on Al_2_O_3_ coated Si substrates. As a good and homogenous DNA hybridization was verified by fluorescence microscopy, this functionalization protocol was chosen for the further study on the functional chips with Al_2_O_3_ passivated NN-FETs.

### 3.3. Chemical Composition after DNA Hybridization on Al_2_O_3_ Passivated Si NN-FETs

**Figure 4:**
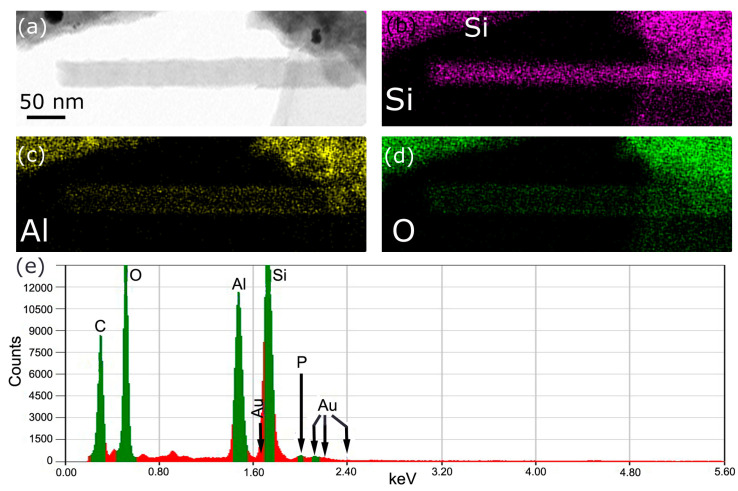
Chemical mapping and quantitative analysis of the elements by STEM EDS of an Al_2_O_3_ passivated Si NN FET after GOPS-based functionalization process. (**a**) Bright field TEM micrograph of a Si NW passivated with Al_2_O_3_. (**b**) Silicon map. (**c**) Aluminum map. (**d**) Oxygen map. (**e**) Quantitative analysis of the elements (Au in the results comes from residues of the metal contacts).

The chemical distribution of elements within and at the surface of NWs was highlighted by STEM EDS (Figure 4). This technique allowed us to obtain an elemental mapping and quantitative analysis on Al_2_O_3_ covered Si NN–FETs after TBA–15 hybridization step. For the elemental mapping, several single NWs have been studied, whereas the quantitative analysis has been conducted on NW assembly to increase the signal of elements in a low concentration, such as phosphorous. A typical bright field image shows a view of one of the single Si NW studied (Figure 4a). The elemental mapping is coherent with the core-shell structure of the Al_2_O_3_ passivated Si NW: the presence of Si within the NW is clearly seen (Figure 4b) while the presence of Al and O is particularly seen at the NW surface (Figure 4c,d). The phosphorous amount is too low on one single NW to have significant information on the mapping. However, the quantitative analysis (Figure 4e) confirms the presence of this element, which is proof that DNA strands are present at the surface of the NWs. This last result indicates that the NNs were efficiently biomodified.

### 3.4. Surface Morphology Evolution at the Main Steps of the Biomodification Process

**Figure 5:**
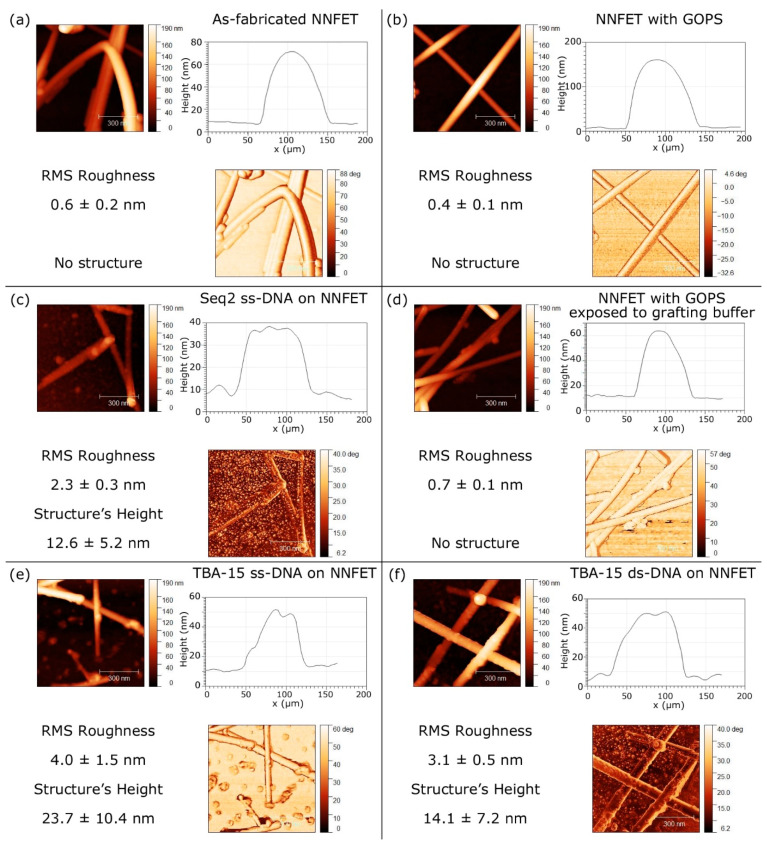
AFM analyses on Al_2_O_3_-passivated Si nanonet FETs at different steps of functionalization. (**a**) Bare Al_2_O_3_ passivated NN–FET. (**b**) After GOPS silanization. (**c**) After Sequence 2 probe grafting. (**d**) After GOPS silanization and exposition to the grafting phosphate buffer (without DNA probe). (**e**) After TBA–15 probe grafting. (**f**) After TBA–15 hybridization with complementary DNA (cTBA target).

Al_2_O_3_-passivated Si NN–FETs were biofunctionalized using the optimized UV-assisted GOPS-based functionalization protocol described above. The evolution of surface morphology was studied using AFM measurements after each main step of the biofunctionalization process to better understand the modification in the surface morphology. Results are reported in Figure 5. For the Al_2_O_3_ passivated Si NN–FET surface, the steps studied were: (i) at the end of the technological protocol (Figure 5a), (ii) after the GOPS silanization (Figure 5b), (iii) after Sequence 2 probe grafting (see Table 1) (Figure 5c), (iv) after TBA–15 probe grafting (See Table 1) (Figure 5e); (v) after TBA–15 hybridization with its complementary DNA target cTBA (See Table 1) (Figure 5f). We also studied the impact of the presence of a phosphate buffer on the GOPS-silanized surface (Figure 5d). The analyzed buffer was the one used to dilute the DNA probes at the grafting step.

In the absence of DNA on the sample surface (Figure 5a,b,d), the latter is flat and the NWs exhibit a smooth shape, as shown on the line profiles, which are almost cylindrical apart from the bottom due to convolution with the AFM tip. Due to such convolution, only the height of the objects is considered in the analysis. For the fabricated devices (Figure 5a), the substrate exhibits an RMS roughness value of 0.6 nm (σ = 0.2 nm). After GOPS deposition (Figure 5b), the shape of the NW is not significantly modified, while the substrate roughness shows a slight decrease to 0.4 nm (σ = 0.1 nm). This slight decrease seems to indicate that the GOPS molecules flatten the natural irregularities of the Al_2_O_3_-covered surface. This also suggests that the GOPS process does not significantly affect the NNs and substrate morphology. Moreover, dipping the sample in the phosphate buffer does not modify the surface morphology (Figure 5d), demonstrating that no crystallized salts remain on the surface after the rinsing step.

As expected, grafting DNA probe on the NN-FET device has a strong impact on the surface morphology (Figure 5c,e). First, it can be noticed that new structures appear both on the substrate and on the nanonet, whether the TBA–15 probe (Figure 5e) or Sequence 2 probe (Figure 5c) are grafted, but their geometries are quite different from one another.

In the case of the TBA–15 probe (Figure 5e), the surface roughness increases significantly to 4.0 nm (σ = 1.5 nm) and the mean height of the structures is 23.7 nm (σ =10.4nm). Besides, in the case of Sequence 2 probe (Figure 5c), the mean height of the structures is reduced by a factor of two, i.e., 12.6 nm (σ = 5.2nm), with a substrate surface RMS roughness of 2.3 nm (σ = 0.3 nm). Such a difference is quite surprising, as both DNA sequences have more or less the same length (see Table 1). However, it is known that guanine-rich nucleic acid sequences, such as TBA–15, can form four-stranded nucleic acid structures named G-quadruplexes. This configuration contains a planar, tetrameric arrangement of guanines, which in turn can be stacked on top of each other, thanks to π–π interactions, being stabilized by monovalent cations, such as K^+^ and Na^+^ [29,30,31,32]. These structures may build different types of multimers, such as wires, stacks of intramolecular structures, and interlocked dimers and trimers [33,34]. Therefore, the significant height observed in the case of the TBA–15 probe could correspond to stacks of G-quadruplexes TBA structures. On the contrary, the Sequence 2 probe cannot form G-quadruplex structures, which could indicate that the irregularities observed in the AFM images correspond to individual DNA probe molecules grafted on the surface. The dimensions found for this sequence are larger than expected, as the theoretical length of a 20-base DNA is about 6.8 nm [35]. However, different factors could affect the results obtained. In the case of oligonucleotide AFM analysis, the different level of hydration of the molecules may play an important role [36]. It is known that a layer of water surrounding the DNA molecules can interact with the tip, resulting in the formation of a meniscus that can lead to an overestimation of the measured height [37].

After the TBA–15 hybridization (Figure 5f), both the surface roughness (3.1 nm, σ = 0.5 nm), and the height of the structures (14.1 nm, σ = 7.2 nm) are lower compared to previous values obtained for the TBA–15 probe (Figure 5e). In this case, the molecules on the surface would correspond to double-stranded DNA, which must present a linear conformation for the hybridization to occur, explaining closer morphology to that of the Sequence 2 probe. (Figure 5c).

### 3.5. Electrical Characterization

#### 3.5.1. Impact of Grafted DNA Probes on FET Electrical Properties

NN FET-based biosensors rely on the field effect. The adsorption of charged species on the surface of the transistor channel modifies the surface charge which influences the density of free carriers within the channel. For example, the adsorption of negative charges on the surface of a P-type channel attracts holes to the surface. The aptamer and DNA sequences used in this work are negatively charged molecules, because of phosphate groups. In solution, counter ions are present to preserve electroneutrality, but when the DNA strands are grafted at the surface of a semiconductor and that sample is dried, electroneutrality is expected from a free carrier reorganization inside the semiconductor.

**Figure 6:**
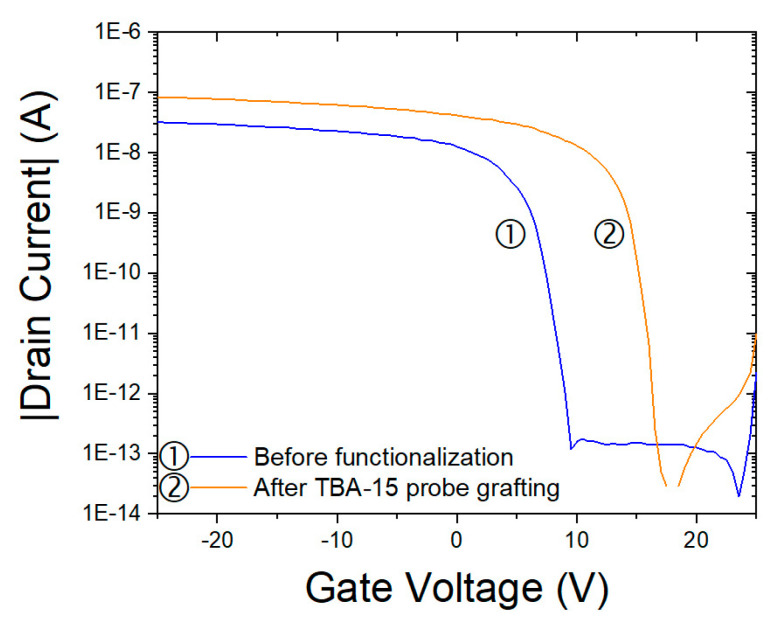
Transfer characteristics obtained for an L = 100 µm, W = 100 µm NN–FET at a drain voltage of V_ds_ = −2 V. The device was located in the quarter Q2 (see Figure 1): ➀ before biofunctionalization process and ➁ after TBA–15 probe grafting.

According to this, after the grafting of the TBA–15 probe or of the Sequence 2 probe, (see Table 1) on the P-channel surface of the nanonet, the transfer characteristic of the transistor—drain current as a function of gate voltage—should be shifted to more positive gate voltages to offset the negative charges brought by the probes. Figure 6 displays an example of the impact of the TBA–15 grafting on the electrical characteristics of a Si NN–FET. The main impact on the characteristics is the change in the transistor drain current at a given gate voltage and the threshold voltage shift.

Before further studying the electrical behavior of the biosensor when in contact with the protein, our aim with this study is to understand exactly the formation of the biosensor and the impact of the various components on the electrical properties of the Si NN-FETs. Notably, as we were able to test numerous devices (around 80) with various geometries, different DNA sequences (TBA–15 or Sequence 2), or solely exposed to the buffer solution, we noticed that the impact on the electrical properties is not so simple, suggesting that several mechanisms for electroneutrality are in competition. We also noticed that the results obtained are highly dependent on the experimental protocol. Particularly, the transfer characteristics obtained after probe grafting are time dependent. It is therefore fundamental to understand the role and the impact of each parameter of the protocol, in order to determine those that will allow us to obtain stable and reproducible experiments. As a consequence, in the following we described in detail the experimental results and the conclusions we draw from our study of electrical Si NN-FET properties based on numerous devices and the impact of DNA grafting on these properties.

#### 3.5.2. Precautions in the Experimental Protocol

**Figure 7:**
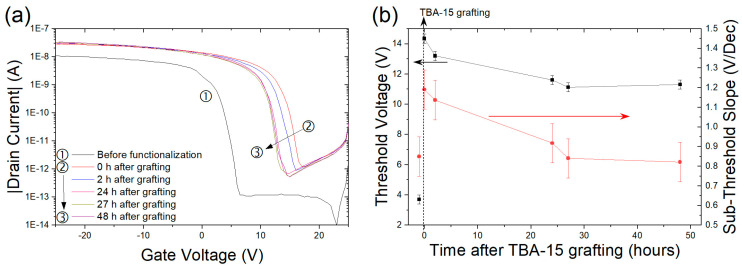
(**a**) Transfer characteristics of a L = 100 µm, W = 100 µm Si NN–FET before functionalization and at different times after TBA–15 grafting. The drain voltage V_ds_ was set at −2 V. (**b**) Sub-threshold slope (SS) (red circles) and threshold voltage (Vth) (black squares) variation for the different times analyzed. Points before time = 0 correspond to the electrical characterizations before TBA–15 probe grafting.

**Electrical properties shift in time after probe grafting**. First, the stability of the transfer characteristics after TBA–15 probe grafting was studied. Figure 7a displays the typical behavior of the transfer characteristic as a function of elapsed time after probe grafting and subsequent exposure to ambient conditions. Irrespective of the channel geometry, after probe grafting the transfer curve is first shifted towards positive voltages with a sub-threshold slope degradation, then it takes 24 h to recover the initial sub-threshold slope and stabilize the threshold voltage, V_th_ (Figure 7b). For 100% of the tested devices, a positive shift of ΔV_th_, varying between 5 and 15 V, is observed upon TBA–15 grafting. As a consequence, it is important to wait for stabilization before acquiring initial transfer characteristics of the functionalized devices.

**Figure 8:**
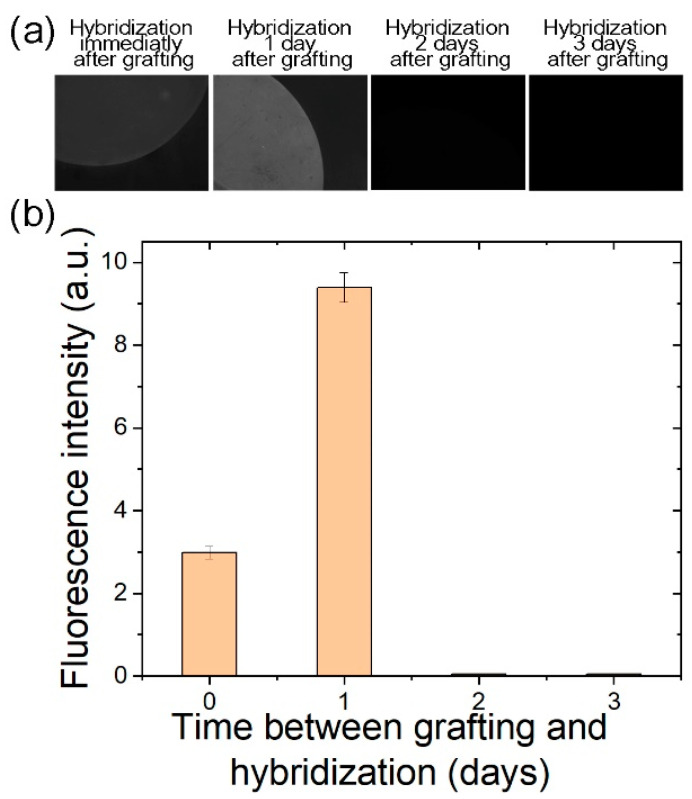
Fluorescence intensity as a function of delay between TBA–15 probe grafting and hybridization when Si/SiO_2_ (100 nm) substrates functionalized by UV-assisted GOPS protocol are kept in ambient conditions. (**a**) Typical epifluorescence microscopy images when the hybridization with the complementary strand (cTBA) was conducted at different times after the TBA–15 grafting (immediately after, 1 day, 2 days, and 3 days after). (**b**) Fluorescence intensity, as measured on fluorescence micrographs, as a function of delay between TBA–15 probe grafting and hybridization. For all conditions, three fluorescent spots on the substrate were analyzed giving an average fluorescence intensity value and error bars corresponding to the minimum and maximum fluorescence intensity found for each sample.

**Hybridization capacity in time.** As described above, after probe grafting, it takes 24 h to stabilize the electrical properties, suggesting modifications in the surface properties. Therefore, it is important to check the impact of time on the grafted TBA–15 probe. For this purpose, we studied its hybridization capacity in time. To gain more sensitivity in fluorescence measurements, this experiment was conducted on Si/SiO_2_ (100 nm) substrates biofunctionalized by UV-assisted GOPS protocol, which were kept in ambient conditions (room temperature in air), and the hybridization with its complementary strand (cTBA) was conducted at different times after the TBA–15 grafting (immediately after, 1 day, 2 days, and 3 days after). The results reported in Figure 8, where the fluorescence evolution is shown as a function of time between grafting and hybridization, indicate that there is an optimum for hybridization after 1 day. However, for a longer waiting time, hybridization is not efficient anymore. Such a result is then quite surprising, as we know from our previous works that fluorescent biosensors are highly stable after probe deposition, as shown by fluorescence observations. In particular, by studying hybridization capacity in time of the Sequence 2 probe, we demonstrate that hybridization with its complementary strand is still efficient after more than one year of storage in air (see Appendix C). As the Sequence 2 probe is still functional for hybridization after a long period of time, we guess that such a difference between the two oligonucleotides arises from the particular sequence of the TBA–15, which is able to form characteristic G-quadruplex structures and has potential to form complex multimers such as wires [33,34]. As the stability of the multimers increases with dehydration [38], one can imagine that once stable wires have been formed, it would then be difficult to achieve the hybridization with the DNA complementary strand. Moreover, such dehydration could also explain the evolution of the electrical characteristics observed in Figure 7. According to our results (Figure 8), after 2 days in air, hybridization is no longer favored over G-quadruplex structure or multimer formation. Such concurrent behavior between hybridization and multimer formation has already been demonstrated for TBA–15 [39]. However, as suggested in this reference, when subjected to sodium-only buffer incubation, the TBA–15 hybridization is favored over G-quadruplex formation. In our case, the hybridization buffer was composed of different ions, including potassium, which facilitates the formation of the G-quadruplex structures, thus reducing the possibility of hybridization taking place.

Therefore, for further analysis, the buffer composition (K^+^ and Na^+^ ions) and the time between TBA–15 probe grafting and target recognition should be chosen according to the objective of the study, depending on whether the aim is to detect the TBA–15 hybridization with its complementary strand, or the thrombin detection, which would be favored with the stabilization of G-quadruplex structures.

#### 3.5.3. Detailed Study of the Grafting Step

**Figure 9:**
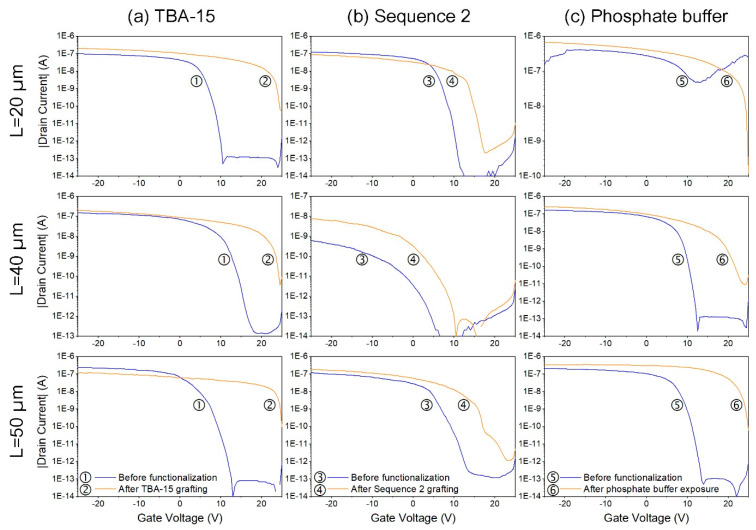
Impact of: (**a**) TBA–15 probe, (**b**) Sequence 2 probe and (**c**) grafting phosphate buffer on transfer characteristic for different channel geometries after 24 h of exposure in ambient conditions. The drain voltage V_ds_ was set at −2 V.

The grafting step has been studied with the aim to understand the impact of the surface environment on the Si NN–FETs. In particular, the surface environment was modified either by probe grafting (TBA–15 and sequence 2) or by immersion in a solution, the phosphate buffer. So, to ensure homogeneity in electrical devices, each quarter of a chip (Figure 1d) was treated in a different way at the grafting step: Q2 and Q4 were functionalized with TBA–15 probe (see Table 1), Q3 was functionalized with Sequence 2 probe (see Table 1) and Q1 was exposed solely to the grafting phosphate buffer, which is the solution used to solubilize the probes at the grafting step, but in this case no DNA probe was added to the solution. Figure 9 displays the transfer characteristic for each case and for three different channel geometries, as fabricated and 24 h after the grafting step or the exposure to the phosphate buffer, in ambient conditions. Considering the as-fabricated devices, one can notice that increasing the channel length improves the reproducibility from one device to another. Such a phenomenon arises from the nanonet properties as previously shown [13]. Indeed, increasing the channel length results in a better averaging effect of the network so that the overall dispersion of the transistor parameters decreases when the channel length increases. For devices exposed to the phosphate buffer, a negligible effect of the buffer on the transfer characteristic would have been welcome in order to guarantee the selectivity of the future sensor. However, the first important observation is that, irrespective of the case, even when no DNA was grafted on the NN (Figure 9c), the threshold voltage is shifted towards more positive values. This means that the surface modification with DNA is not the only mechanism responsible for variations in surface charge and that another phenomenon should be involved. This effect can be explained simply on the basis of the pH of the solution. Indeed, the chosen phosphate buffer is fixed at pH = 8.5. According to the literature, the point of zero charge (PZC) of alumina is between 5.4 and 9.5 [40], and more precisely PZC of thin film alumina deposited by atomic layer deposition (ALD) is around 8.0 [41]. Moreover, it appears that GOPS silanization does not significantly change the PZC [42]. Then, if pH > PZC, the surface is negatively charged. As a consequence, in our configuration, with the pH = 8.5, the channel surface should be negatively charged when in contact with the phosphate buffer, implying therefore a charge reorganization within the channel, exactly as the DNA grafting does, and explaining the ΔV_th_ shift observed toward positive values.

A second observation is that the ΔV_th_ shift is higher for the TBA–15 probe (Figure 9a) than for Sequence 2 probe (Figure 9b). According to the literature, DNA bases exhibit different values of electronegativity, presenting the following order in a decreasing way: Thymine: 4.80 eV, Guanine: 4.68 eV, Cytosine: 4.51 eV, Adenine: 4.38 eV [43]. TBA–15 probe is composed exclusively of Thymine (T) and Guanine (G) bases (see Table 1), which are the most electronegative bases, while the Sequence 2 probe is composed of a combination of the four different bases (Table 1). Then, with TBA–15 being a more electronegative sequence, it would have the ability to generate a higher change in the surface charge, resulting in a larger ΔV_th_ shift.

To conclude, the obtained results demonstrate that the positive V_th_ shift has two independent origins: the negative alumina surface charges (case of phosphate buffer in Figure 9c) and the negative charges brought by the two kinds of DNA probes grafted on the Si NN (Figure 9a,b). Further experiments are needed to complete these preliminary results. Notably, it will be important to quantify and enlighten the respective contributions of each observed effect on the electrical properties. On the one hand, regarding the impact of buffer solution on the surface device charge, and with the aim of eliminating this parameter, additional analysis should be carried out, using a buffer at pH = 8, which corresponds to the PZC of thin film alumina deposited by atomic layer deposition. In this case, only the DNA charges would be involved in the electrical characteristic modification on the Al_2_O_3_-passivated Si NN–FETs. On the other hand, regarding the differences in the DNA molecules characteristics (TBA–15 and Sequence 2), a deeper investigation of their surface distribution and conformation should be performed. Once these experiments are carried out, it will be possible to characterize and study the thrombin recognition effect on the electrical properties of the Si NN–FET devices.

## 4. Conclusions

On the basis of a study on numerous Si NN–FETs (around 80 with various geometry) functionalized with DNA probes (TBA–15 and Sequence 2), we evidenced important experimental parameters that have to be taken into account in view of optimal reproducibility in experimental results. In the first part of our work, we determined the best silanization process to be used with electrical devices. Subsequently, we optimized the GOPS-based grafting protocol by using 20 min UVA irradiation prior to the DNA probe drop casting, resulting in a homogeneity enhancement of the surface DNA distribution. Then, by studying carefully the surface morphology of the Si NN–FETs by AFM, we showed that the base sequence in the DNA strand drastically impacted its structure on the surface. This can be attributed to different factors such as (1) the formation of DNA multimers in the case of guanine-rich nucleic acid sequences, which can be stabilized by sodium, or more favorably by potassium ions and (2) the variation in hydration levels of the molecules that depend on the bases in the sequence, which modifies the interaction between the AFM tip and the surface and, hence, impacts the measurements.

Regarding the stability of the devices in ambient conditions, we demonstrated that the electrical properties after the TBA–15 probe grafting are time dependent, which should be linked to hydration evolution. After probe grafting, the transfer characteristics are shifted toward positive voltages with a sub-threshold slope degradation, then it takes 24 h to recover the initial sub-threshold slope and stabilize the threshold voltage.

Furthermore, in the biofunctionalization control experiments carried out by the hybridization of TBA–15 probe with its complementary sequence, it was observed that the hybridization capacity is also time dependent. The fact that no fluorescence was observed when hybridization was performed 2 days after the grafting step can be explained by the formation of the multimers from G-quadruplex structures, which could have been stabilized by the dehydration of the structures as time elapsed and then reinforced by the presence of potassium ions in the hybridization buffer. Thus, for further analyses, it will be necessary to choose the buffer composition and the time between TBA–15 probe grafting and target recognition according to the objective of the study, depending on whether the aim is to detect the TBA–15 hybridization with its complementary strand, or the thrombin detection, which would be favored with the stabilization of G-quadruplex structures.

Finally, a detailed study of the aptamer grafting step by means of electrical characterizations allowed us to demonstrate that not only the DNA grafted on the surface implies a threshold voltage positive shift, but also the immersion in the buffer solution, whose pH is higher than the surface PZC. Moreover, we have shown that the shift in V_th_ is dependent on the base sequence of the grafted DNA strand, due to the variation of the bases’ electronegativity.

This work is a preliminary but critical step towards studying and understanding the impact of thrombin recognition on the electrical properties of nanonet-based devices. In particular, we can summarize the points of attention as follows:

1. The pH of the solutions used must be equal to the PZC of the surface materials or the surface material must be chosen so that its PZC is equal to the pH of the solutions.
2. In the case of dry detection, the rate of hydration of the surface has an impact on the electrical response and must therefore be controlled by the storage conditions and/or the time before measurement.
3. In the case where multimer formation may occur, it is important to choose the ions in solution according to the desired structure, single-strands or multimers.
4. It should be noted that the sequence of bases in the DNA strand plays a role on the electrical response of the device.

## Figures and Tables

**Table 1:** Summary of the DNA sequence used in this work.

Name	Sequence	Function	Elongated Length (nm)
TBA–15	^5′^GGTTGGTGTGGTTGGTTTTTT^3′^–NH_2_	Probe	7.1
Sequence 2	^5′^NH_2_-TTTTTGATAAACCCACTCTA^3′^	Probe	6.8
cTBA	Cy3-^5′^CCAACCACACCAACC^3′^	Target	5.1
c-Sequence 2	Cy3-^5′^CATAGAGTGGGTTTATCCA^3’^	Target	6.5

**Figure A1:**
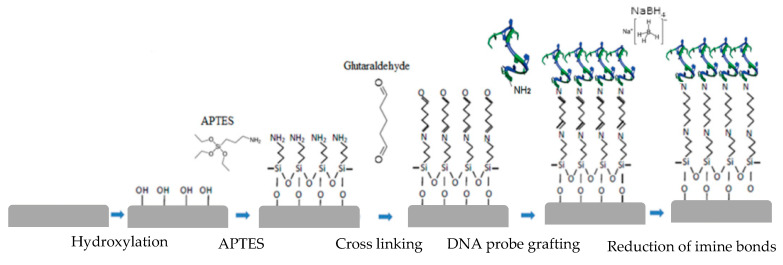
APTES-based functionalization protocol for covalent DNA probe grafting.

**Figure A2:**
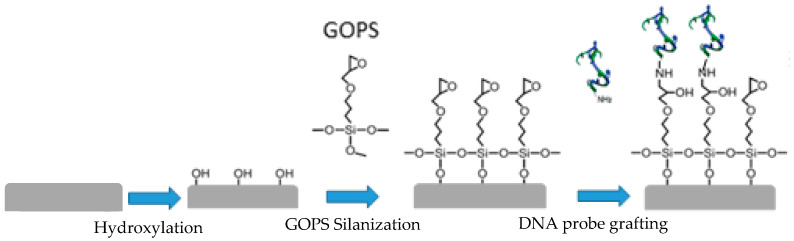
GOPS-based functionalization protocol for covalent DNA probe grafting.

**Figure A3:**
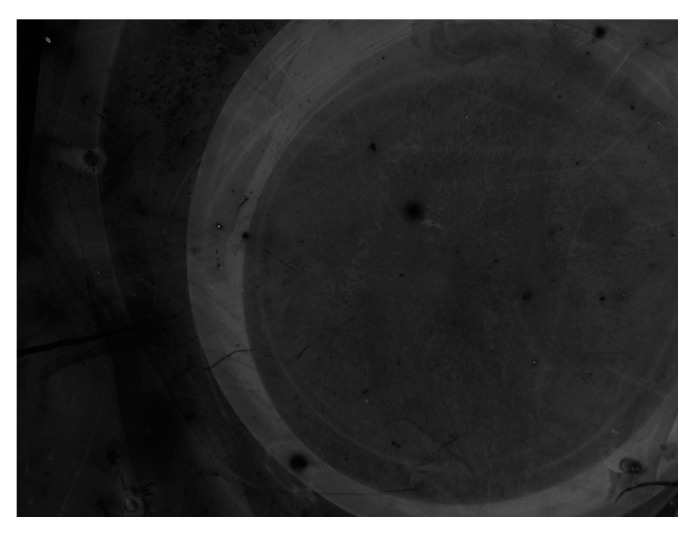
Image by epifluorescence microscopy corresponding to the hybridization of the Sequence 2 probe with its complementary strand, performed 17 months after the probe grafting. The analysis was carried out on Si/SiO_2_ (100 nm) substrates functionalized by UV-assisted GOPS protocol, using an exposure time of 3 s.

## Appendix A. Amino-Silane (APTES)-Based Biofunctionalization Protocol

APTES-based DNA probe grafting process begins with the activation of the surface by generating hydroxyl bonds through the action of an O_2_/air plasma. Subsequently, 3-Aminopropyl triethoxysilane (APTES) vapor phase silanization in anhydrous conditions is carried out at 80 °C for 1 h, followed by an ethanol rinsing and a heat treatment at 110 °C for 1 h to remove the remaining water, resulting in the formation of a silane self-assembled monolayer on the surface of the samples. This stage is followed by a cross-linking step using a 10% aqueous glutaraldehyde solution for 90 min, followed by deionized water rinsing. This dialdehyde allows one to bind the amines at the end of the silanized surface and the amine-terminated DNA probes dissolved in a phosphate buffer solution. The sample is left to react overnight at room temperature and ambient conditions. In order to obtain a more chemically stable structure, the double bond of the imines is reduced to a single bond by using a 0.09 M NaBH_4_ aqueous solution for 1 h. Finally, the samples are rinsed with a 0.2% sodium dodecyl sulphate aqueous solution and deionized water. The whole process is summarized in the Figure A1.

## Appendix B. Epoxy-Silane (GOPS)-Based Biofunctionalization Protocol

The GOPS DNA probe grafting process consists of three stages. Initially the activation of the surface is carried out by the creation of hydroxyl groups due to exposure to an O_2_/air plasma. Silanization is then carried out by depositing (3-Glycidyloxypropy)trimethoxysilane (GOPS) in vapor phase and in anhydrous conditions at 100 °C for 1 h, followed by an ethanol rinsing and a heat treatment at 135 °C for 1 h in order to remove the remaining water, giving rise to a silane self-assembled monolayer on the surface of the samples. The epoxy-terminated surface allows a direct covalent attachment of NH_2_-terminated DNA probes on the surface via ring-opening reaction on the epoxy group. The sample is left to react for 1 h at room temperature and ambient conditions, subsequently it is placed in a humidity chamber at 60 °C for 30 min and then kept overnight at room temperature and ambient conditions. Finally, the samples are rinsed with a 0.2% sodium dodecyl sulphate aqueous solution and deionized water. The whole process is summarized in the Figure A2.
